# Long-Term Consumption of Platycodi Radix Ameliorates Obesity and Insulin Resistance via the Activation of AMPK Pathways

**DOI:** 10.1155/2012/759143

**Published:** 2012-07-05

**Authors:** Chae Eun Lee, Haeng Jeon Hur, Jin-Taek Hwang, Mi Jeong Sung, Hye Jeong Yang, Hyun-Jin Kim, Jae Ho Park, Dae Young Kwon, Myung-Sunny Kim

**Affiliations:** Biogeron Technology Research Group, Korea Food Research Institute, Baekhyon-dong 516, Bundang-gu, Songnam, Gyongki-do 463-746, Republic of Korea

## Abstract

This study was designed to evaluate the effects and mechanism of Platycodi radix, having white balloon flower (*Platycodon grandiflorum for. albiflorum (Honda) H. Hara*) on obesity and insulin resistance. The extracts of Platycodi radix with white balloon flower were tested in cultured cells and administered into mice on a high-fat diet. The Platycodi radix activated the AMPK/ACC phosphorylation in C2C12 myotubes and also suppressed adipocyte differentiation in 3T3-L1 cells. In experimental animal, it suppressed the weight gain of obese mice and ameliorated obesity-induced insulin resistance. It also reduced the elevated circulating mediators, including triglyceride (TG), T-CHO, leptin, resistin, and monocyte chemotactic protein (MCP)-1 in obesity. As shown in C2C12 myotubes, the administration of Platycodi radix extracts also recovered the AMPK/ACC phosphorylation in the muscle of obese mice. These results suggest that Platycodi radix with white balloon flower ameliorates obesity and insulin resistance in obese mice via the activation of AMPK/ACC pathways and reductions of adipocyte differentiation.

## 1. Introduction

Obesity has become a major public health problem with increasing prevalence and it induces an insulin resistant state in adipose tissue, liver, and muscle. Insulin resistance is a strong risk factor for the development of type 2 diabetes and the common cause for other metabolic diseases, such as hypertension, coronary artery disease, and strokes [1–3]. There is a considerable need for safe therapeutic agents that can reduce the risk of obesity-induced metabolic dysfunctions. The range of natural products and foods with the potential benefits for obesity continue to expand.

Platycodi radix (PR), the root of *Platycodon grandiflorum* has been used as a food and a traditional medicine for bronchitis, asthma in Korea. In clinical practice of oriental medicine, it is often used in combination with basic herbal medicines for detoxification in the treatment of upper respiratory infection, acute and chronic bronchitis [4]. Recently, it has shown the novel pharmacological potentials for treating metabolic diseases, such as hyperlipidemia and diabetes [5–8]. It has shown that antiobesity and antihypolipidemic effects of platycodin saponins in animal models are due to the inhibition of intestinal absorption of dietary fat [5, 6]. It showed not only the protective effects on fatty liver via suppression of pancreatic lipase activity [8], as well as acceleration of energy expenditure; but also the hepatoprotective effects on EtOH-induced hepatotoxicity in animal models [8–10]. However, the mechanism by which Platycodi radix helps to respond in obesity and insulin resistance still remains to be elusive.

The adenosine monophosphate- (AMP-) activated protein kinase (AMPK), which senses the cellular energy charge, a metabolic master switch. When activated by ATP depletion, it turns off ATP-consuming processes, such as fatty acid synthesis, cholesterol synthesis, and gluconeogenesis, while turning on catabolic pathways that generate ATP, such as glycolysis, *β*-oxidation, and glucose uptake. Many lines of evidence had suggested that activation of AMPK increases fatty acid oxidation by inactivating acetyl Coenzyme A (ACC) and lowering the concentration of malonyl coenzyme A; an inhibitor of carnitine palmitoyl transferase (CPT)-1 in muscle [11–14]. We found that the stimulation of cultured myotubes with the extracts of platycodi radix induces the phosphorylation of AMPK and ACC. Since the alteration of impaired function of skeletal muscle in obesity is the primary cause for insulin resistance, we hypothesized that Platycodi radix may affect the alteration of some key regulators in obesity and insulin resistance. In this study, we demonstrated the effects and mechanism of Platycodi radix having white balloon flower on obesity and insulin resistance.

## 2. Materials and Methods

### 2.1. Chemicals and Reagents

C2C12 cells and 3T3-L1 fibroblasts were purchased from American Type Culture Collection (ATCC, MD). Dulbecco's modified Eagle's medium (DMEM) and fetal bovine serum (FBS) were from WelGENE biopharmaceuticals (Daegu, Korea). Isobutylmetyl-xanthine (IBMX), dexamethasone (DEX), and insulin were purchased from Sigma Chemical (St. Louis, MO, USA).

Antibodies that recognize phosphorylated AMPK Thr172 and acetyl-CoA carboxylase (ACC) Ser79 were purchased from Cell Signaling Technology (Beverly, MA) and *β*-actin was purchased from BETHYL Laboratories (Montgomery, TX). Horseradish peroxidase-conjugated anti-rabbitIgG were obtained from Enzo life sciences (Farmingdale, NY). Bicinchoninic acid (BCA) and 5-aminoimidazole-4-carboxamide ribofuranoside (AICAR) were purchased from Sigma Chemical (St. Louis, MO) and compound C was purchased from CalBiochem (San Diego, CA).

### 2.2. Preparation of White Balloon Flower Platycodon Radix

The root of platycodi radix with white balloon flower (WBF) was grown for 3-4 years in Jeju Island and was subsequently used in the experiment. Dried and ground powder of Platycodi radix was supplied by Hangirim JK Milk thistle research institute (Jeju, Korea). One hundred grams of powdered platycodi radix was extracted with 900 mL of 70% ethanol by shaking for 24 hrs at 25°C and repeated 2 more times. The precipitates were removed by centrifuging at 8,000 ×  g for 30 minutes (Beckman, Brea, CA). Finally, the supernatants were lyophilized by freezing dryer (Il Shin, Korea). Total extracts were dissolved in dimethyl sulfoxide (DMSO) for cell treatment and dissolved in water for animal diets.

### 2.3. Cell Culture and Differentiation

C2C12 and 3T3-L1 cells were maintained in DMEM supplemented with 10% FBS and 100 unit antibiotics at 37°C in a 5% CO_2_ atmosphere. For differentiation into myotubes, C2C12 cells were cultured in DMEM and supplemented with 2% horse serum for 3 days and stimulated with indicated concentration of the extracts. For adipocyte differentiation, 3T3-L1 cells were cultured for 2 days in 24-well plates until showing 100% confluency [15]. To induce differentiation, confluent 3T3-L1 cells (designated as day 0) were replaced by DMEM containing 10% FBS, 10 *μ*g/mL insulin, 0.5 *μ*M DEX and 0.5 mM IBMX for 40 hrs. After induction of differentiation, the medium was changed with 10% FBS/DMEM supplemented with 10 *μ*g/mL of insulin and refreshed every 2 days for 7 days. Differentiated adipocytes were used to evaluate lipid accumulation with Oil red O staining.

### 2.4. Oil Red O Staining

Adipocyte differentiation was monitored under a microscope and by staining the cells with Oil Red O [16]. At the end of differentiation, cells were washed gently with PBS and fixed in 3.7% paraformaldehyde for 10 min. Oil red O staining solution (3 : 2 mixture of 0.2% Oil Red O-Isopropanol solution and water) was added to the cells and kept at room temperature for 1 hr and then cells were washed with deionized water before being photographed. For quantification of Oil red O uptake, cells were incubated with isopropanol for 10 min, RT. The absorbance was measured at 510 nm (VERSA Max Microplate Reader, Molecular Device, Concord, Canada).

### 2.5. Whole Cell Extract and Western Blotting

Cell monolayers were washed in ice-cold PBS and solublized in lysis buffer (1% Triton X-100, 1 mM EDTA, 50 mM Tris (pH 7.6), 150 mM NaCl, 1 mM NaF) containing 0.2% protease inhibitor cocktails, 1 mM phenylmethylsulfonyl fluoride (PMSF), and the phosphatase inhibitor. The frozen muscles from animals were homogenized in 3 volumes of cold RIPA lysis buffer. Samples were boiled for 10 min at 95°C after quantification of protein concentrations by BCA method. Lysates were subjected to 10% SDS/PAGE and transferred to the nitrocellulose membrane. The membrane was blocked with 0.02% Tween 20/Tris-buffered saline (TBS) containing 5% nonfat dried milk. The blot was incubated with pAMPK, pACC, and *β*-actin antibodies and then with secondary antibodies conjugated to horseradish peroxidase. The bands were visualized by enhanced chemiluminecence (ECL) reagents (DaeilLab Service, Seoul, Korea).

### 2.6. Animals and Diets

All protocols for animal use were approved by the Institutional Animal Care and Use Committee of the Korea Food Research Institute (KFRI) and were in accordance with Korea National Institutes of Health guidelines. Six-week old male C57BL/6 mice obtained from Charles River Korea (Seoul, Korea) were housed at KFRI at a constant temperature (22–26°C) under light/dark cycles of 12 hrs per day. Mice had access to autoclaved water and pellet food ad libitum. Mice were fed with 10% kcal fat diet (D12450B), 60% kcal high fat diet (D12492, Research Diets, New Brunswick, NJ), 0.1% and 1% platycodi radix in 60% kcal high fat diet for 10 weeks. Body weight and food intakes were measured every week. Serum was prepared from blood collected from the eyes of mice just before sacrifice, and the harvested gastrocnemius muscles were immediately put into liquid nitrogen and stored at −70°C until analysis. The blood glucose concentration was monitored in venous blood drawn from the tail vein with glucometer (AccuCheck, Roche, Basel, Switzerland) after overnight fasting. Plasma insulin levels were measured by a quantitative sandwich enzyme immunoassay kit (ALPCO Diagnostics, Salem, NH).

### 2.7. Glucose Tolerance Test

The intraperitoneal glucose tolerance test was performed at 8 weeks of the experiment. Following 15 hrs of fasting, blood glucose levels were measured at the initial time. And then, the mice were injected intraperitoneally with 1 g D-glucose/kg of body weight. The blood glucose levels were monitored at 30, 60, 90, 120, 150 min after glucose injection. The area under the curve (AUC) was also calculated based on the blood glucose curves.

### 2.8. Histological Examination

Epididymal white adipose tissue and liver removed from mice were fixed in 10% formalinand embedded in paraffin. The sections were dehydrated in graded concentrations of alcohols, embedded in paraffin, and stained with hematoxylin and eosin (H&E). Sections were observed under a microscope at a magnification of ×200 (Nikon, Japan).

### 2.9. Measurement of Circulating Mediators

Triglyceride (TG) and total cholesterol (T-CHO) reagent were purchased from Asan Pharmaceutical (Seoul, Korea). TG and T-CHO in serum were measured by the manufacturer's protocol. Cytokines leptin, resistin, and MCP-1 were measured by sandwich ELISA method. The kits for leptin and MCP-1 were obtained from PeproTech (Rocky Hill, NJ) and resistin was from R&D systems (Minneapolis, MN). Precoated plates with capture antibody for overnight were incubated with serums and each standard for 2 hrs at room temperature. Next, biotin-labeled detection antibody and avidin horseradish peroxidase conjugate were added to the plate and incubated at room temperature. Tetramethylbenzidine liquid solution was added to the plate and incubated at room temperature. The reaction was then stopped with 1 N H_2_SO_4_. The absorbance was measured at 450 nm in VERSA Max Microplate Reader (Concord, Canada).

### 2.10. Statistical Analysis

Data are presented as mean ± SEM of at least three independent experiments performed in triplicate. Statistical differences of the results were evaluated by the unpaired student's *t*-test. *P* value <0.05, 0.01 was considered to be significant.

## 3. Results

### 3.1. The Effects of PR on AMPK/ACC Phosphorylation and Adipogenesis

**Figure 1:**
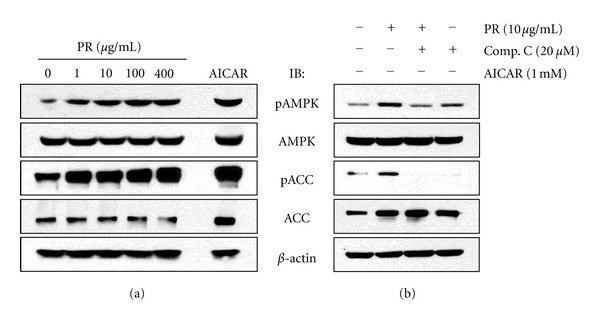
Platycodi Radix activates AMPK and ACC phosphorylation in C2C12 myotubes. (a) Differentiated C2C12 myotubes were stimulated with various concentration (1, 10, 100, 400 *μ*g/mL) of PR for 1 hr. (b) Compound C (20 *μ*M) was pretreated for 30 min before stimulation of 10 *μ*g/mL PR. The cell lysates were analyzed by Western blotting for pAMPK (Thr172) and pACC (Ser79). The anti-*β*-actin blot was visualized as an internal control.

We first examined the effects of PR extracts on the phosphorylation of AMPK in differentiated C2C12 myotubes. Activation of AMPK*α* was assessed by measuring phosphorylated AMPK (pAMPK) levels. As shown in Figure 1(a), treatment of WBF PR extracts for 1 hr strongly increased the phosphorylation of AMPK in a dose-dependent manner and brought to a maximum increase at 10 *μ*g/mL. Simultaneously, WBF PR extracts increased the phosphorylation of ACC, a major substrate of AMPK in malonyl Co A synthesis and fatty acid oxidation. AICAR, which mimics the activating effect of AMP on AMPK, was used as a positive control. To confirm the PR-stimulated activation of AMPK, we pretreated with 20 *μ*M compound C, an AMPK-specific inhibitor for 30 min and followed by PR treatment. Compound C blocked the phosphorylation of AMPK as well as ACC upon PR stimulation (Figure 1(b)), indicating that the WBF PR stimulates AMPK/ACC phosphorylation pathway.

**Figure 2:**
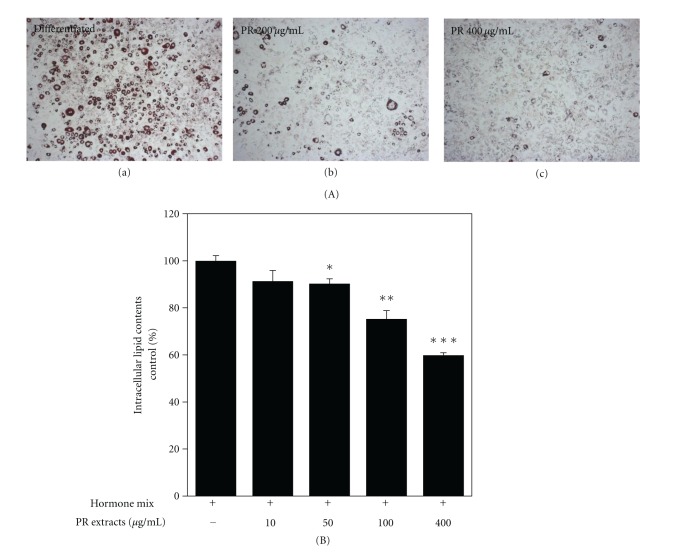
Platycodi Radix inhibits adipogenic differentiation and intracellular lipid accumulation. 3T3-L1 cells were propagated to confluence and then differentiation was induced by treatment of hormone mixture (10 mg/mL of insulin, 0.5 mM of DEX, and 0.5 mM IBMX for 40 hrs and kept with 10 mg/mL of insulin and/or 10, 50, 100, 400 *μ*g/mL PR for 7 days. Lipid accumulation was measured by Oil Red O staining. (A) Representative photomicrographs of lipid accumulation. (a) Fully differentiated cells for 7 days, (b) 200 *μ*g/mL PR extracts treated cells, (c) 400 *μ*g/mL PR extracts treated cells. (B) Relative intracellular lipid contents. It is considered as significant data when *P* value is <0.05 between fully differentiated cells and PR-treated groups. Data are expressed as mean ± SEM of triplicate experiments.; *P < 0.05; **P < 0.01; ***P < 0.001.

We also observed the inhibitory effects of PR on adipogenic differentiation and intracellular lipid accumulation (Figure 2). Treatment of PR in 3T3-L1 cells decreased adipocyte differentiation in a dose-dependent manner as indicated by the reduction in Oil Red O incorporation. Four hundreds *μ*g of PR significantly suppressed lipid accumulation to 60% levels of fully differentiated cells. However, PR extracts did not show any cytotoxic effects, as determined by lactate dehydrogenase (LDH) assay of the supernatants (Data not shown). Adipocyte differentiation may have been efficiently blocked by PR extracts.

### 3.2. The Effects of PR on Body Weight Changes and Insulin Resistance

**Figure 3:**
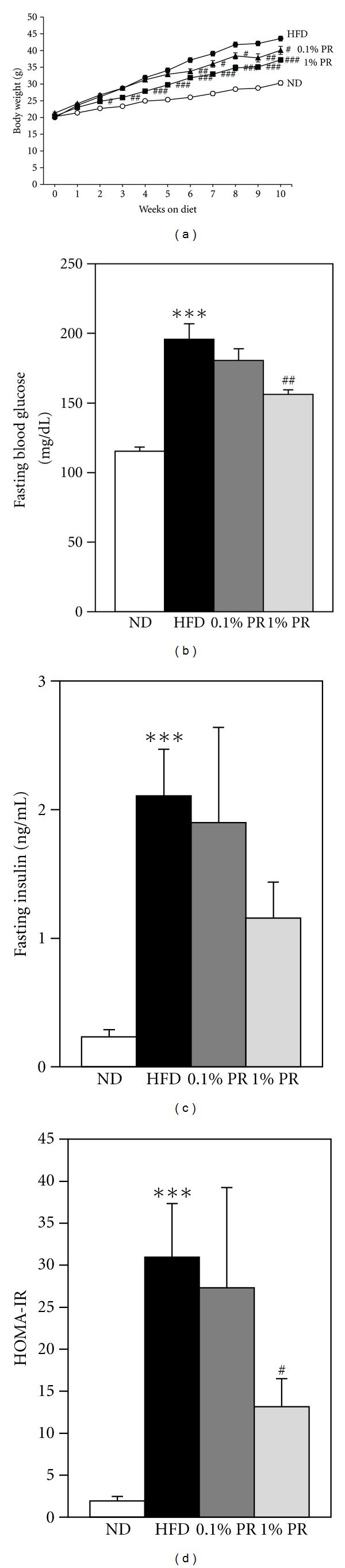
Administration of Platycodi Radix ameliorates weight gain and insulin resistance. Mice were fed 10% kCal fat diet (D12450B), 60% kCal high fat (D12492) diet (Research Diets, New Brunswick, NJ), 0.1% and 1% WBF Platycodon radix in 60% kCal for 10 weeks. Body weight and food intakes were measured every week. (a) Body weight changes for 9 weeks, fed status. (b) Fasting blood glucose. (c) Fasting serum insulin. (d) HOMA-IR. Data are mean ± SEM. *P < 0.05;  **P < 0.01; ***P < 0.001 on ND versus HFD. ^#^ *P* < 0.05; ^##^ *P* < 0.01; ^###^ *P* < 0.001 on HFD versus WBF PR-treated group.

To evaluate the potential effects of PR extracts on obesity and insulin resistance in mice, C57BL/6 mice were fed with ND, HFD, and 0.1%, 1% PR formula with HFD (PR-HFD) for 10 wks. The body weight of mice on HFD was significantly higher than that of mice on ND. Administration of 1% PR-HFD slowed down the weight gain at 2 wks and was sustained for 10 wks (Figure 3(a)). Low dosages of PR administration also showed the reduction of weight gain. Food intake was significantly higher on a high-fat diet group than the normal diet group. However, PR administration did not show any effect on food intake, which means the decreased weight gain, was not from changed food intake (Table 1).

**Figure 4:**
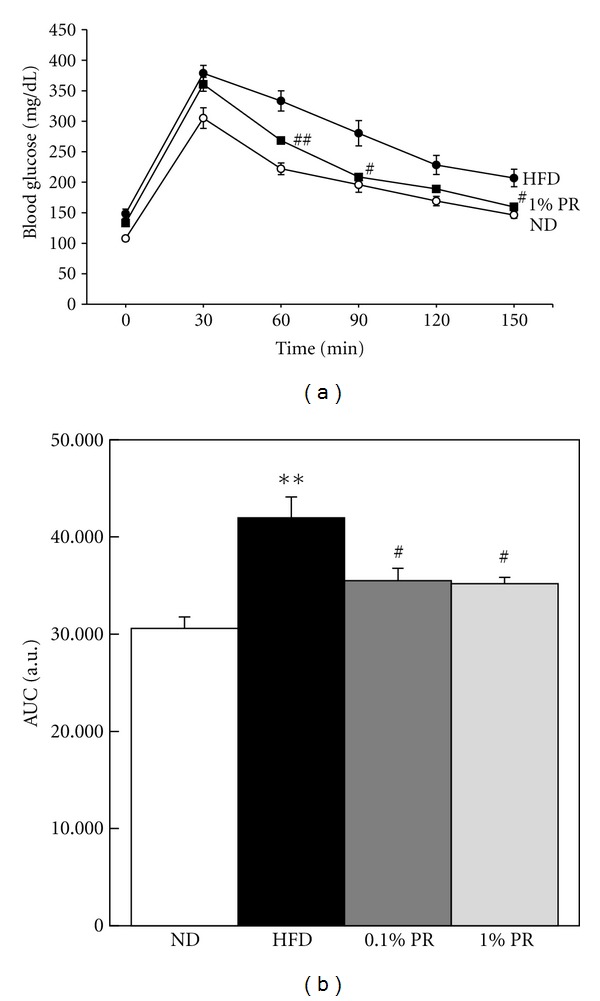
Administration of Platycodi Radix improves glucose intolerance of obese mice. Intraperitoneal glucose tolerance test was performed at 8 wks of the experiment. Following 15 hr fasting and glucose injection, blood glucose levels were monitored at 0, 30, 60, 90, 120, 150 min. (a) Glucose curves. (b) Area under the curve (AUC). *P < 0.05;**P < 0.01 on ND versus HFD. ^#^ *P* < 0.05; ^##^ *P* < 0.01 on HFD versus WBF PR-treated group.

PR administration ameliorated fasting plasma levels of glucose, insulin, and homeostasis model assessment of insulin resistance (HOMA-IR, Figures 3(b), 3(c), and 3(d)). Plasma glucose levels during ipGTT were significantly increased in HFD more than in ND. Whereas the clearance of blood glucose levels was delayed in obese mice, it was attenuated in mice fed PR-HFD (Figure 4(a)). The AUC values of plasma glucose levels during the ipGTT were significantly increased on HFD and this increase was attenuated in PR-HFD mice (Figure 4(b)). These results suggest that glucose intolerance seen in HFD-fed mice was significantly ameliorated by administration of PR. Taken together; we suggest that PR is effective on the reduction of body weight gain as well as glucose intolerance.

### 3.3. Circulating Mediators, Fat, Liver Tissues

**Figure 5:**
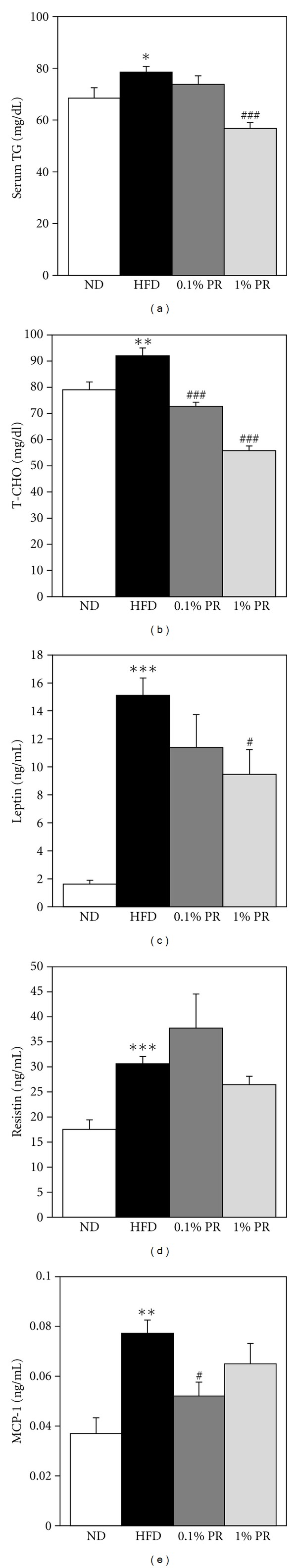
Administration of Platycodi Radix reduces serum levels of TG, T-CHO, and intracellular cytokines. Mice were fed 10% kCal fat diet (ND), 60% kCal high fat (HFD) diet, 0.1% and 1% PR in 60% kCal HFD for 10 weeks. Serum was prepared from blood collected from the eyes of mice just before sacrifice. (a) TG. (b) Total cholesterol. (c) Leptin. (d) Resistin. (e) MCP-1.

Plasma total triglyceride (TG) and cholesterol (T-CHO) levels were also significantly increased by HFD, but were ameliorated on PR-HFD (Figures 5(a) and 5(b)). The plasma adipokines including leptin, resistin on PR-HFD were ameliorated on PR-HFD (Figures 5(c) and 5(d)). The inflammatory cytokines, MCP-1 level was also shown a decreased tendency (Figure 5(e)).

**Figure 6:**
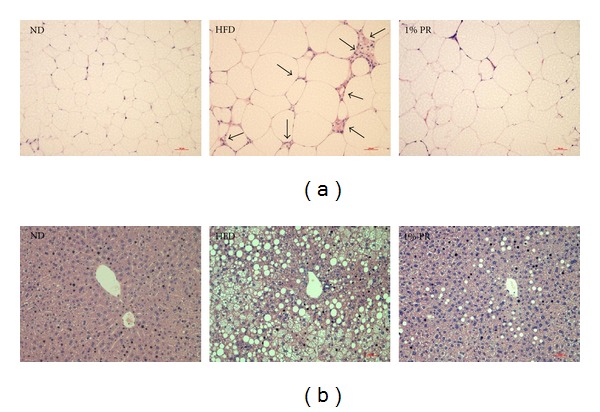
Administration of Platycodi Radix reduces adipose tissue expansion and liver lipid accumulation. Epidydimal white adipose tissue and liver removed from mice were fixed in 10% formalin and embedded in paraffin. The sections were stained with hematoxylin-eosin. Representative photomicrographs of H&E stained: (a) adipose tissue, (b) liver tissue.

Since the local tissues, such as adipose tissue, liver, and muscle are the primary target organs of metabolism in obesity and insulin resistance, we tried to observe its phenotypes. Obese mice on HFD significantly increased the weight of visceral adipose and liver tissues (Table 1). PR administration significantly reduced the weight of both tissues. Parallel to visceral adipose tissue weight, adipocyte size distribution was shifted to a smaller cell size in PR supplemented groups, when compared to HFD mice (Figure 6(a)). The infiltrated cells with crown-like structures were also significantly decreased on PR-HFD (Figure 6(a)). The lipid accumulation in the liver also significantly decreased on PR-HFD (Figure 6(b)). However, plasma glutamic-oxaloacetic transaminase (GOT) and Glutamic-pyruvic transaminase (GPT) levels were not altered in each group, which shows nontoxicity of PR to live and other organs (data not shown). The concentration of GOT and GPT, the enzymes that exist in the liver and other organs, in the blood is maintained at constant levels due to normal cellular destruction. If many cells of the liver and other organs are damaged, these enzymes are secreted to serum [17].

Taken together, these data indicate that PR administration ameliorated impaired circulating mediators, as well as phenotypes of local tissue in obese mice.

### 3.4. The Effects of PR on AMPK/ACC Phosphorylation in Muscle

**Figure 7:**
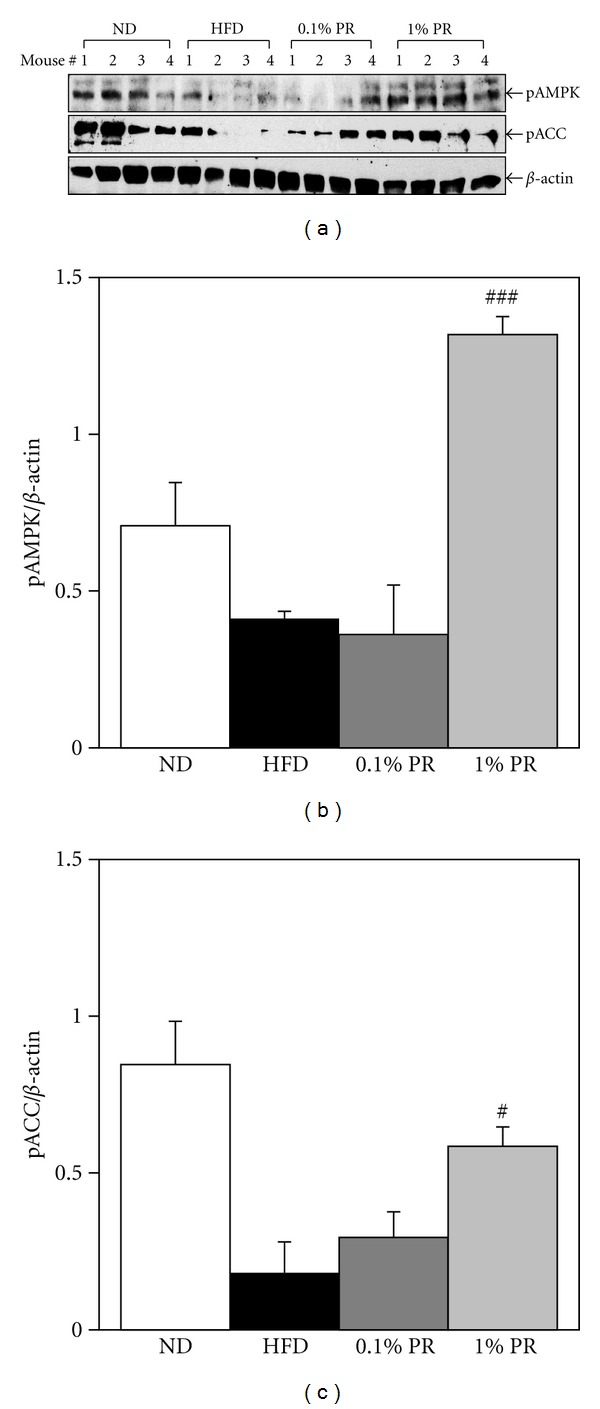
Administration of Platycodi Radix activates the ACC/AMPK phosphorylation in the muscle of obese mice. Mice were on 10% kCal ND, 60% kCal HFD, 0.1% and 1% PR in 60% kCal HFD for 10 weeks. Protein was extracted from individual frozen gastrocnemius muscles. (a) It is immunoblotted with pAMPK (Thr172) and pACC (Ser79) antibodies. The anti-*β*-actin blot was visualized as an internal control. (b) pAMPK and (c) pACC levels were quantitated by image densitometry and normalized by *β*-actin expression. ^#^ *P* < 0.05; ^##^ *P* < 0.01 on HFD versus WBF PR-treated group.

To validate the stimulation of AMPK pathways in mice, individual muscle tissue was saved for immunoblotting. We randomly chose 4 mice muscles of each group and blotted with pAMPK and pACC; a common indicator of AMPK activity. Although there is individual variation, the mice on HFD showed a decreased pattern of AMPK phosphorylation, when compared with ND mice (Figures 7(a) and 7(b), *P* = 0.07). PR-treated mice exhibited significantly recovered pAMPK protein levels in gastrocnemius muscles, whereas internal control *β*-actin was not changed. When quantified by image analysis, it shows that the ratio of p AMPK levels to internal control was recovered by 1% PR administration significantly (Figure 7(b)). Consistent with the alteration in pAMPK, the pACC levels were recovered in muscles of PR-treated mice (Figure 7(c)). Our results show that PR administration recovered impaired phosphorylation levels of pAMPK*α* and pACC in the muscle of mice fed a high-fat diet.

## 4. Discussion

Platycodi radix, the root of *Platycodon grandiflorum* has been used as a traditional medicine for bronchitis, asthma, pulmonary tuberculosis, hyperlipidemia, and hypercholesterolemia. It contains triterpenoid saponins, carbohydrates, and fibers [18, 19]. Recently, many studies have focused on the identification of an individual saponin from platycodi radix that has biological activities including anti-inflammation, antihyperlipidemia, antiobesity, and apoptosis [20–25]. It also has shown that the polysaccharides from *P. grandiflorum* specifically activate B cells and macrophages, but not T cells [26].

In our analysis, the crude saponin fraction in the whole extracts was only around 1.5% in Platycodi radix (data not shown). Since most of the people consume the whole root as food or ground powder in Korea, we tried to use the whole extracts of Platycodi radix in animal study to evaluate the function and mechanism. We tried to feed two doses of PR to mice, based on daily intake of humans. A lower dosage, 0.1% PR/diet was based on daily consumption of 100 mg/Kg body weight in traditional medicine. We also administered a higher dosage, 1% PR/diet, to show the extended function in experimental animals. In high-fat-induced obese mice, long-term administration of both doses of PR whole extracts significantly reduces body weight gain, as well as insulin resistance (Figure 3). However, PR administration did not show any changes on food intake and liver toxicity. The glucose tolerance seen in HFD-fed mice was significantly ameliorated by the administration of PR (Figure 4). It is followed by the reduction of circulating lipid mediators, including TG and total cholesterol, markers of obesity. Administration of PR reduced adipokines, such as leptin and resistin, which was increased in obese mice (Figure 5). MCP-1, an inflammatory marker in obesity was also reduced by PR administration. It is coincident with previous report, which saponins from PR have shown the anti-inflammatory activites [20, 27–29]. However, the elevated number of circulating leukocytes in obesity was not modulated by PR (data not shown).

In local tissues, such as liver and adipose tissue, PR administration significantly reduced the lipid accumulation and abnormal phenotypical changes (Figure 6). In the same context, it also disrupted the adipocyte differentiation in the adipocyte study (Figure 2). Recently, it is reported that platycodin D of *Platycodon grandiflorum *inhibits adipogenesis through the regulation of Kruppel-like factor 2 (KLF2), an inhibitor of PPAR*γ* and WNT/*β*-catenin pathway [30, 31]. Lee et al. [31] have shown that platycodin D of *Platycodon grandiflorum* activates the WNT/*β*-catenin pathway, leading to upregulation of nuclear *β*-catenin, which inhibits adipogenic differentiation. Though we treated whole extracts of PR, it is sufficiently effective *in vitro*.

An important finding in this study is that Platycodi radix increased the phosphorylation of AMPK in C2C12 myotubes and in the muscle of mice on PR diet (Figures 1 and 7). We also observed the phosphorylation of ACC upon PR stimulation. Blocking of AMPK by compound C, an AMPK-specific inhibitor, disrupted the phosphorylation of AMPK upon PR stimulation (Figure 1(b)). Increasing evidence has revealed that activation of AMPK lowers the concentration of malonyl coenzyme A by phosphorylating and inhibiting ACC, the rate-limiting enzyme in malonyl CoA synthesis [11–14]. The reduced level of malonyl CoA results in impairment of intracellular fatty acid oxidation through inhibition of CPT-1 activities. Thus, ACC gives the potential for modulating both long-chain fatty acid biosynthesis and mitochondrial fatty acid oxidation. This modulation presents opportunities for treatment of both diabetes and obesity [32].

In the muscle of obese mice, we observed the altered phosphorylation of AMPK/ACC (Figure 7). The inhibition of pAMPK*α*, pACC, as well as AMPK*α* activities in skeletal muscle of obese mice was also associated with insulin resistance [33]. Long-term consumption of PR extracts in high fat diet-induced obese mice recovered the altered AMPK/ACC phosphorylation in muscle tissue (Figure 7). Since skeletal muscle is the predominant tissue responsible for insulin-stimulated glucose disposal and a major site of insulin resistance in diabetes [34], this finding gives us the beneficial potentials of PR.

In the liver, Khanal et al. [10] have shown that the root of *platycodon grandiflorum* lead to recover the phosphorylation of AMPK/ACC pathway, which is impaired in alcoholic fatty liver injury of rat. Although they did not show a direct stimulatory effect in cells, they postulated that the saponins from PR inhibit alcohol-dependent TG accumulation in hepatocytes through AMPK-dependent fatty acid oxidation. Combined with the result in C2C12 myotubes, we suggest that the AMPK/ACC-mediated pathway by PR extracts contributes to ameliorate obesity and insulin resistance. Work is in progress to elucidate the regulation of PR on glucose metabolism and insulin resistance. It will increase the basic understanding of the preventive mechanism against obesity-induced insulin resistance.

## 5. Conclusion

In this study, we conclude that long-term consumption of PR in high-fat diet-induced obesity significantly reduces body weight gain, as well as insulin resistance. It is followed by the reduction of circulating mediators, including triglyceride (TG), total cholesterol, leptin, and monocyte chemotactic protein-1 (MCP-1) in obesity. In local tissues, liver and adipose tissue, PR administration significantly reduced the lipid accumulation and abnormal phenotypical changes in both tissues. In muscle cells, it increased the AMPK/ACC phosphorylation parallel to the phosphorylation events in C2C12 myotubes. Taken together, these results suggest that Platycodon radix with white balloon flower ameliorates obesity and insulin resistance in obese mice via, in part, the activation of AMPK/ACC pathway and reduction of adipocyte differentiation.

## Figures and Tables

**Table 1:** Effects of Platycodon radix on weight changes and food intake. Mice were on 10% kCal ND, 60% kCal HFD, 0.1% and 1% WBF Platycodi radix in 60% kCal HFD for 10 weeks. Food intakes were measured every week and averaged.

	ND	HFD	0.1% PG/HFD	1% PG/HFD
Body weight (g)	27.81 ± 0.59	40.90 ± 0.67^∗∗∗^	37.61 ± 1.16^#^	34.94 ± 0.65^###^
Weight gain (g)	7.57 ± 0.64	20.94 ± 0.54^∗∗∗^	16.33 ± 1.01^##^	14.56 ± 0.74^###^
Gonadal fat (g)	0.90 ± 0.07	2.54 ± 0.11^∗∗∗^	2.46 ± 0.16	2.20 ± 0.10^##^
Liver (g)	0.96 ± 0.02	1.26 ± 0.08^∗∗^	1.08 ± 0.08	0.97 ± 0.03^#^
Food intake (g/mouse/day)	3.18 ± 0.06	2.81 ± 0.07^∗∗∗^	2.70 ± 0.09	2.81 ± 0.08
